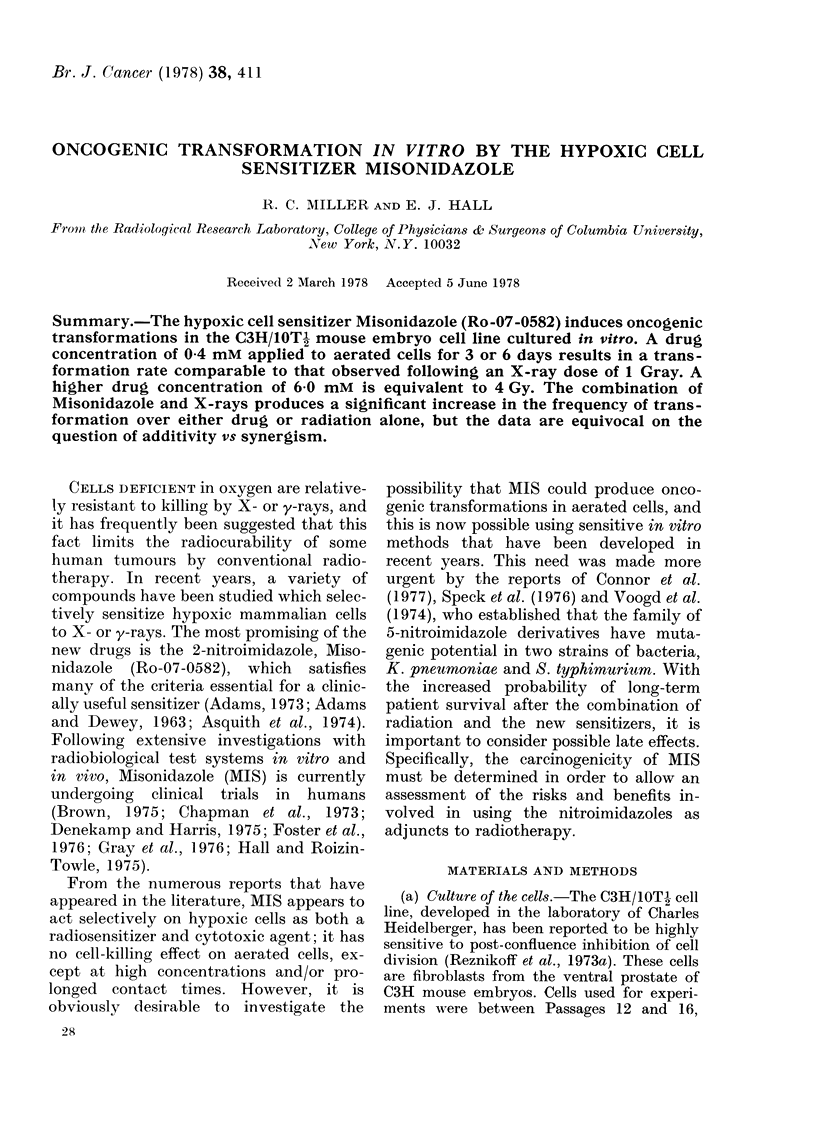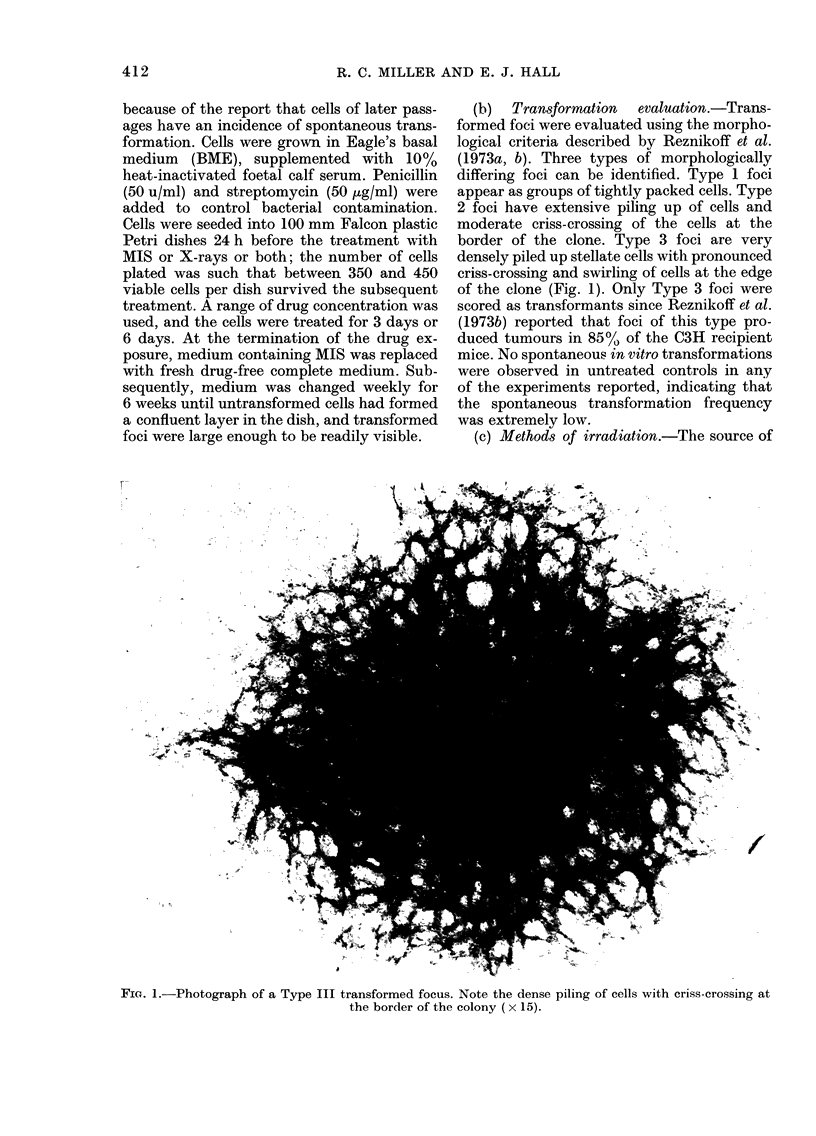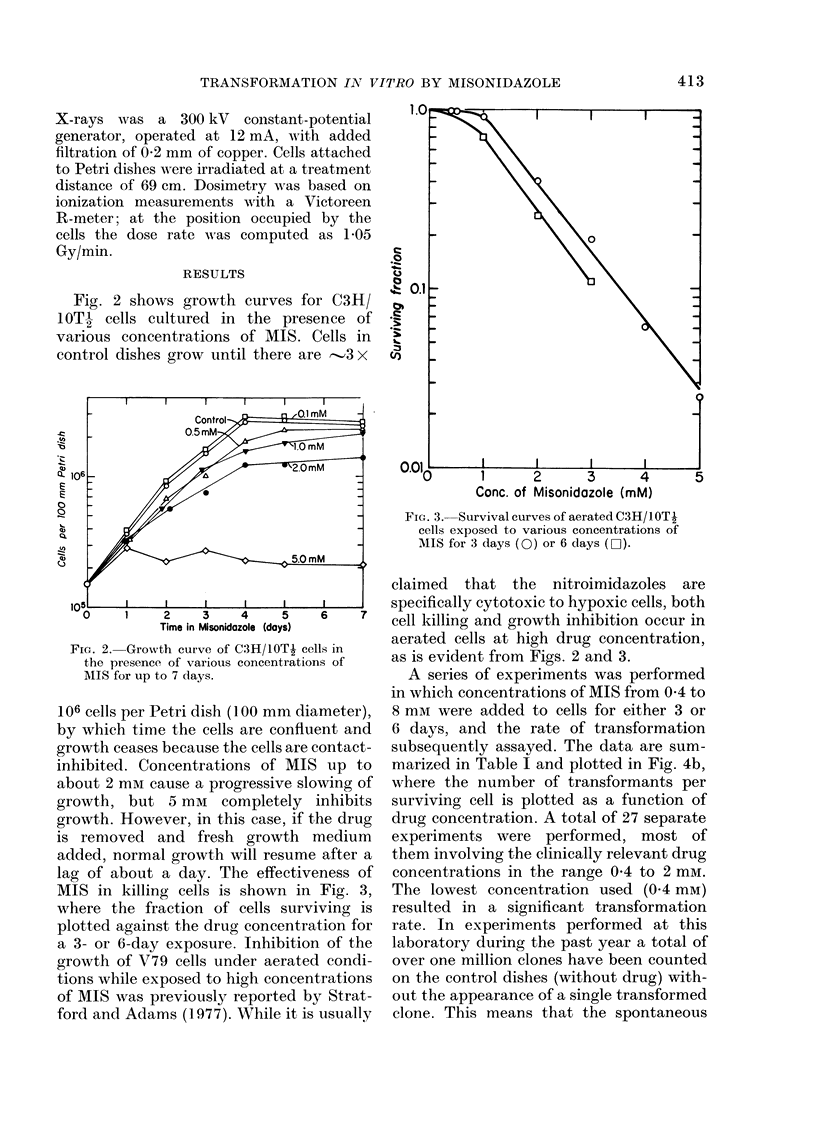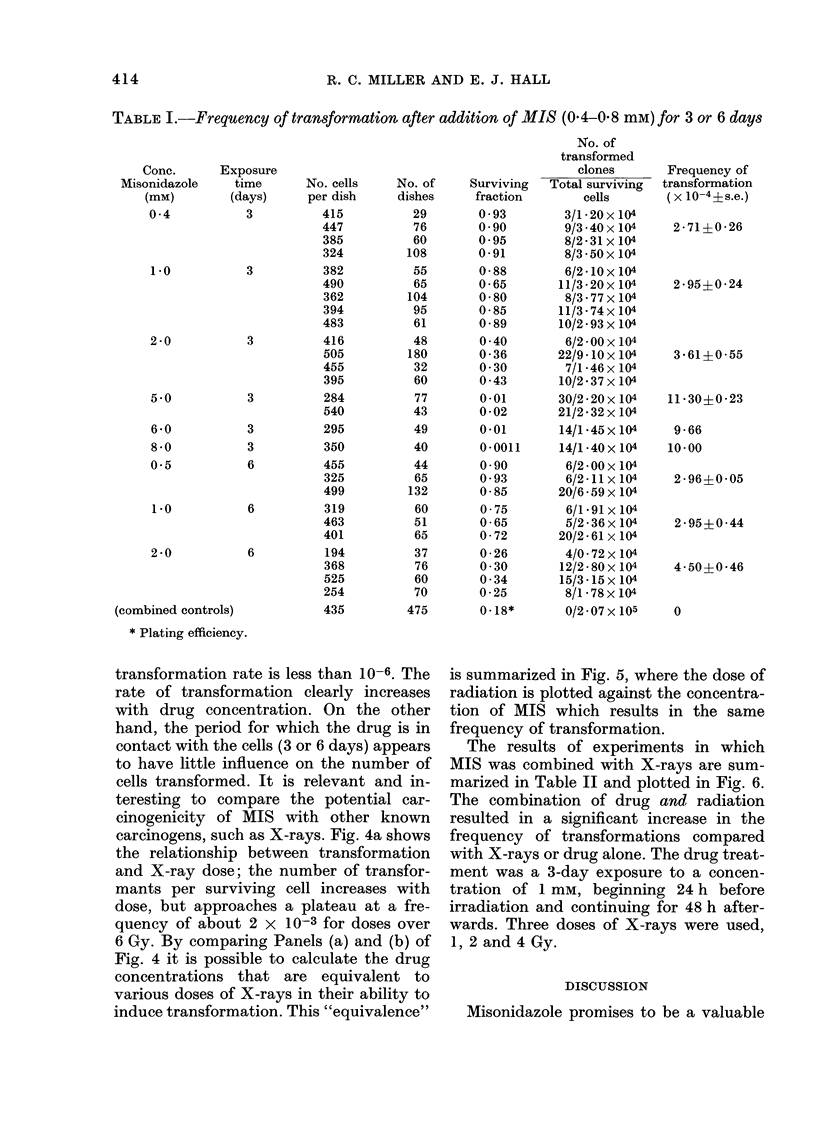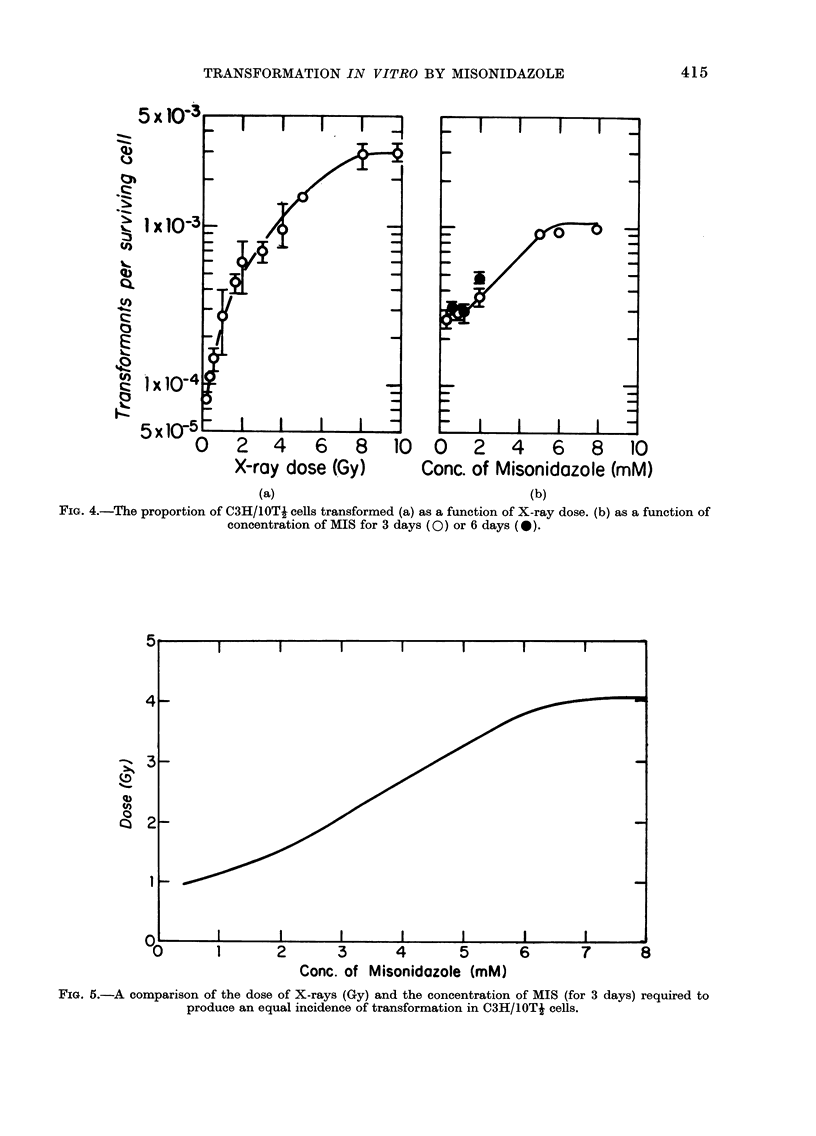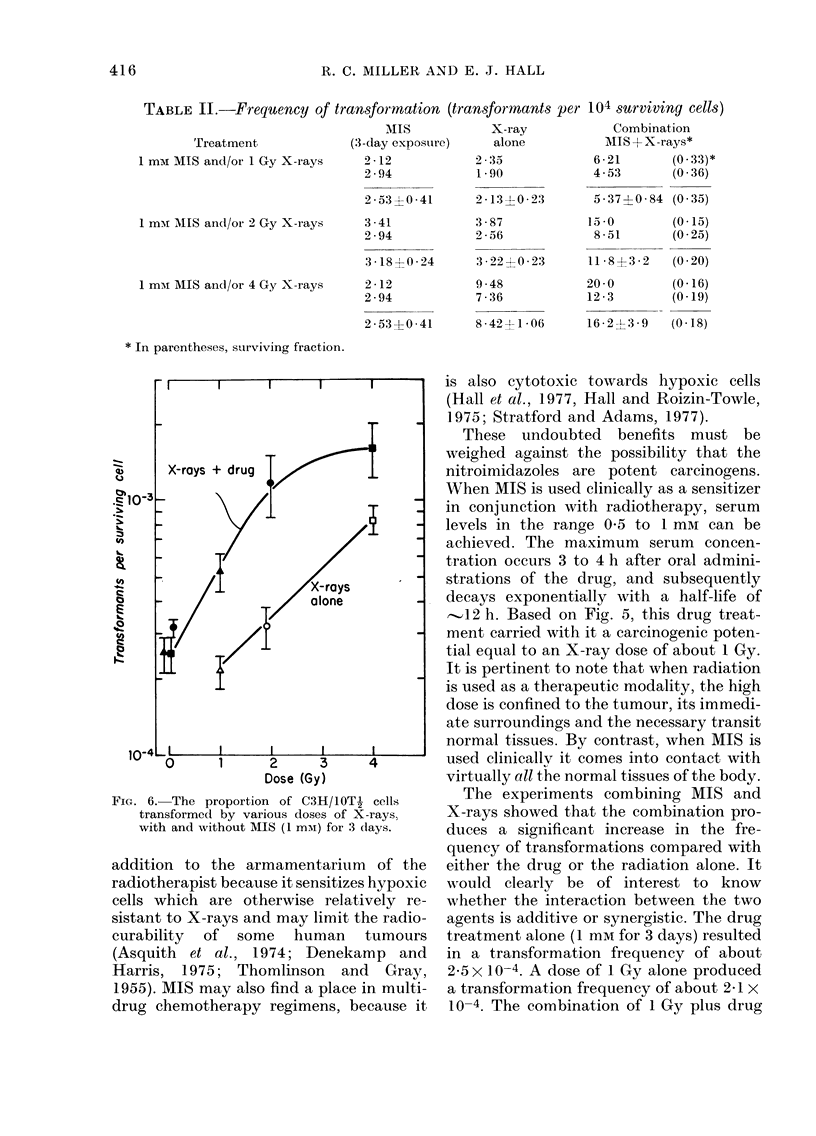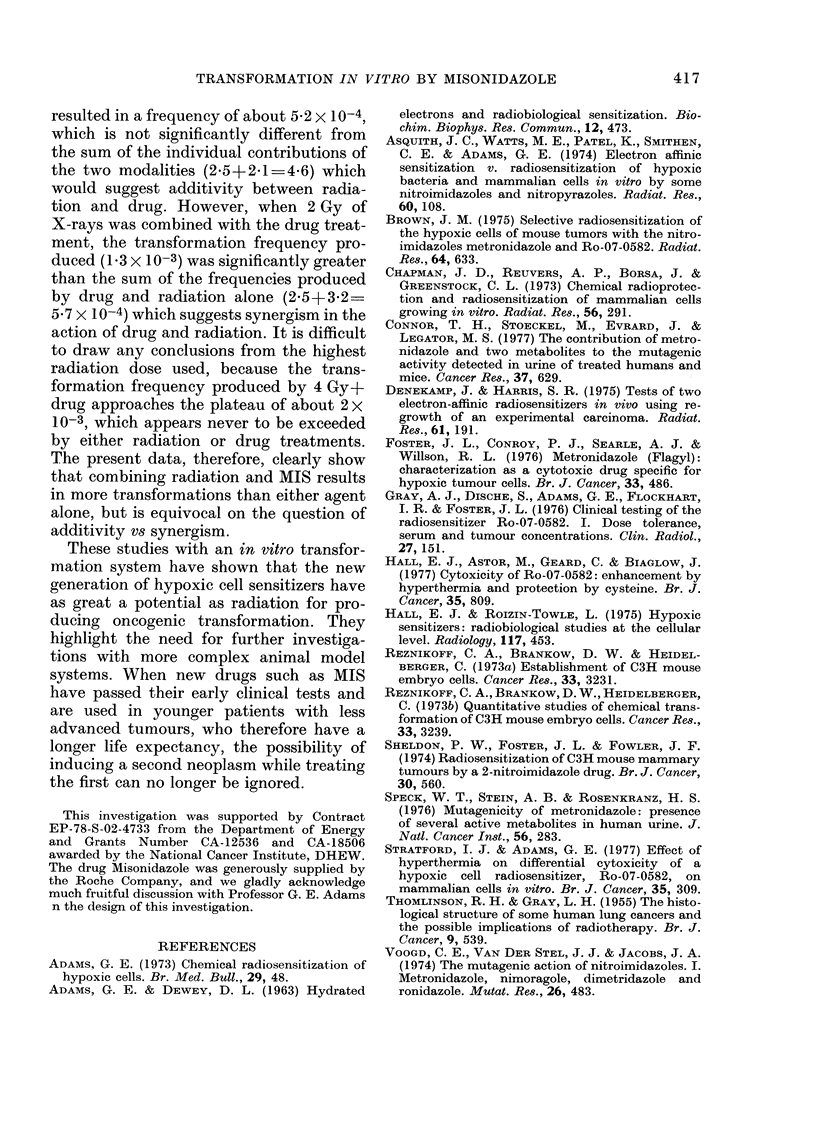# Oncogenic transformation in vitro by the hypoxic cell sensitizer misonidazole.

**DOI:** 10.1038/bjc.1978.222

**Published:** 1978-09

**Authors:** R. C. Miller, E. J. Hall

## Abstract

**Images:**


*Br. J. Cancer* (1978) **38**, 411

### ONCOGENIC TRANSFORMATION *IN VITRO* BY THE HYPOXIC CELL SENSITIZER MISONIDAZOLE

R. C. MILLER AND E. J. HALL

*From the Radiological Research Laboratory, College of Physicians & Surgeons of Columbia University, New York, N.Y.* 10032

Received 2 March 1978 Accepted 5 June 1978

**Summary.—The hypoxic cell sensitizer Misonidazole (Ro-07-0582) induces oncogenic transformations in the C3H/10T½ mouse embryo cell line cultured *in vitro*. A drug concentration of 0·4 mM applied to aerated cells for 3 or 6 days results in a transformation rate comparable to that observed following an X-ray dose of 1 Gray. A higher drug concentration of 6·0 mM is equivalent to 4 Gy. The combination of Misonidazole and X-rays produces a significant increase in the frequency of transformation over either drug or radiation alone, but the data are equivocal on the question of additivity *vs* synergism.**

CELLS DEFICIENT in oxygen are relatively resistant to killing by X- or $\gamma$-rays, and it has frequently been suggested that this fact limits the radiocurability of some human tumours by conventional radiotherapy. In recent years, a variety of compounds have been studied which selectively sensitize hypoxic mammalian cells to X- or $\gamma$-rays. The most promising of the new drugs is the 2-nitroimidazole, Misonidazole (Ro-07-0582), which satisfies many of the criteria essential for a clinically useful sensitizer (Adams, 1973; Adams and Dewey, 1963; Asquith *et al.*, 1974). Following extensive investigations with radiobiological test systems *in vitro* and *in vivo*, Misonidazole (MIS) is currently undergoing clinical trials in humans (Brown, 1975; Chapman *et al.*, 1973; Denekamp and Harris, 1975; Foster *et al.*, 1976; Gray *et al.*, 1976; Hall and Roizin-Towle, 1975).

From the numerous reports that have appeared in the literature, MIS appears to act selectively on hypoxic cells as both a radiosensitizer and cytotoxic agent; it has no cell-killing effect on aerated cells, except at high concentrations and/or prolonged contact times. However, it is obviously desirable to investigate the possibility that MIS could produce oncogenic transformations in aerated cells, and this is now possible using sensitive *in vitro* methods that have been developed in recent years. This need was made more urgent by the reports of Connor *et al.* (1977), Speck *et al.* (1976) and Voogd *et al.* (1974), who established that the family of 5-nitroimidazole derivatives have mutagenic potential in two strains of bacteria, *K. pneumoniae* and *S. typhimurium*. With the increased probability of long-term patient survival after the combination of radiation and the new sensitizers, it is important to consider possible late effects. Specifically, the carcinogenicity of MIS must be determined in order to allow an assessment of the risks and benefits involved in using the nitroimidazoles as adjuncts to radiotherapy.

### MATERIALS AND METHODS

(a) *Culture of the cells.*—The C3H/10T½ cell line, developed in the laboratory of Charles Heidelberger, has been reported to be highly sensitive to post-confluence inhibition of cell division (Reznikoff *et al.*, 1973*a*). These cells are fibroblasts from the ventral prostate of C3H mouse embryos. Cells used for experiments were between Passages 12 and 16, because of the report that cells of later passages have an incidence of spontaneous transformation. Cells were grown in Eagle's basal medium (BME), supplemented with 10% heat-inactivated foetal calf serum. Penicillin (50 u/ml) and streptomycin (50 $\mu$g/ml) were added to control bacterial contamination. Cells were seeded into 100 mm Falcon plastic Petri dishes 24 h before the treatment with MIS or X-rays or both; the number of cells plated was such that between 350 and 450 viable cells per dish survived the subsequent treatment. A range of drug concentration was used, and the cells were treated for 3 days or 6 days. At the termination of the drug exposure, medium containing MIS was replaced with fresh drug-free complete medium. Subsequently, medium was changed weekly for 6 weeks until untransformed cells had formed a confluent layer in the dish, and transformed foci were large enough to be readily visible.

(b) *Transformation evaluation.*—Transformed foci were evaluated using the morphological criteria described by Reznikoff *et al.* (1973*a*, *b*). Three types of morphologically differing foci can be identified. Type 1 foci appear as groups of tightly packed cells. Type 2 foci have extensive piling up of cells and moderate criss-crossing of the cells at the border of the clone. Type 3 foci are very densely piled up stellate cells with pronounced criss-crossing and swirling of cells at the edge of the clone (Fig. 1). Only Type 3 foci were scored as transformants since Reznikoff *et al.* (1973*b*) reported that foci of this type produced tumours in 85% of the C3H recipient mice. No spontaneous *in vitro* transformations were observed in untreated controls in any of the experiments reported, indicating that the spontaneous transformation frequency was extremely low.

(c) *Methods of irradiation.*—The source of

FIG. 1.—Photograph of a Type III transformed focus. Note the dense piling of cells with criss-crossing at the border of the colony (×15).

X-rays was a 300 kV constant-potential generator, operated at 12 mA, with added filtration of 0·2 mm of copper. Cells attached to Petri dishes were irradiated at a treatment distance of 69 cm. Dosimetry was based on ionization measurements with a Victoreen R-meter; at the position occupied by the cells the dose rate was computed as 1·05 Gy/min.

### RESULTS

Fig. 2 shows growth curves for C3H/10T½ cells cultured in the presence of various concentrations of MIS. Cells in control dishes grow until there are ~3 × $10^6$ cells per Petri dish (100 mm diameter), by which time the cells are confluent and growth ceases because the cells are contact-inhibited. Concentrations of MIS up to about 2 mM cause a progressive slowing of growth, but 5 mM completely inhibits growth. However, in this case, if the drug is removed and fresh growth medium added, normal growth will resume after a lag of about a day. The effectiveness of MIS in killing cells is shown in Fig. 3, where the fraction of cells surviving is plotted against the drug concentration for a 3- or 6-day exposure. Inhibition of the growth of V79 cells under aerated conditions while exposed to high concentrations of MIS was previously reported by Stratford and Adams (1977). While it is usually claimed that the nitroimidazoles are specifically cytotoxic to hypoxic cells, both cell killing and growth inhibition occur in aerated cells at high drug concentration, as is evident from Figs. 2 and 3.

FIG. 2.—Growth curve of C3H/10T½ cells in the presence of various concentrations of MIS for up to 7 days.

FIG. 3.—Survival curves of aerated C3H/10T½ cells exposed to various concentrations of MIS for 3 days (○) or 6 days (□).

A series of experiments was performed in which concentrations of MIS from 0·4 to 8 mM were added to cells for either 3 or 6 days, and the rate of transformation subsequently assayed. The data are summarized in Table I and plotted in Fig. 4b, where the number of transformants per surviving cell is plotted as a function of drug concentration. A total of 27 separate experiments were performed, most of them involving the clinically relevant drug concentrations in the range 0·4 to 2 mM. The lowest concentration used (0·4 mM) resulted in a significant transformation rate. In experiments performed at this laboratory during the past year a total of over one million clones have been counted on the control dishes (without drug) without the appearance of a single transformed clone. This means that the spontaneous

TABLE I.—*Frequency of transformation after addition of MIS* (0·4–0·8 mM) *for* 3 *or* 6 *days*

| Conc. Misonidazole (mM) | Exposure time (days) | No. cells per dish | No. of dishes | Surviving fraction | No. of transformed clones / Total surviving cells | Frequency of transformation ($\times 10^{-4} \pm$ s.e.) |
|---|---|---|---|---|---|---|
| 0·4 | 3 | 415 | 29 | 0·93 | $3/1\cdot20\times10^4$ | |
| | | 447 | 76 | 0·90 | $9/3\cdot40\times10^4$ | $2\cdot71\pm0\cdot26$ |
| | | 385 | 60 | 0·95 | $8/2\cdot31\times10^4$ | |
| | | 324 | 108 | 0·91 | $8/3\cdot50\times10^4$ | |
| 1·0 | 3 | 382 | 55 | 0·88 | $6/2\cdot10\times10^4$ | |
| | | 490 | 65 | 0·65 | $11/3\cdot20\times10^4$ | $2\cdot95\pm0\cdot24$ |
| | | 362 | 104 | 0·80 | $8/3\cdot77\times10^4$ | |
| | | 394 | 95 | 0·85 | $11/3\cdot74\times10^4$ | |
| | | 483 | 61 | 0·89 | $10/2\cdot93\times10^4$ | |
| 2·0 | 3 | 416 | 48 | 0·40 | $6/2\cdot00\times10^4$ | |
| | | 505 | 180 | 0·36 | $22/9\cdot10\times10^4$ | $3\cdot61\pm0\cdot55$ |
| | | 455 | 32 | 0·30 | $7/1\cdot46\times10^4$ | |
| | | 395 | 60 | 0·43 | $10/2\cdot37\times10^4$ | |
| 5·0 | 3 | 284 | 77 | 0·01 | $30/2\cdot20\times10^4$ | $11\cdot30\pm0\cdot23$ |
| | | 540 | 43 | 0·02 | $21/2\cdot32\times10^4$ | |
| 6·0 | 3 | 295 | 49 | 0·01 | $14/1\cdot45\times10^4$ | 9·66 |
| 8·0 | 3 | 350 | 40 | 0·0011 | $14/1\cdot40\times10^4$ | 10·00 |
| 0·5 | 6 | 455 | 44 | 0·90 | $6/2\cdot00\times10^4$ | |
| | | 325 | 65 | 0·93 | $6/2\cdot11\times10^4$ | $2\cdot96\pm0\cdot05$ |
| | | 499 | 132 | 0·85 | $20/6\cdot59\times10^4$ | |
| 1·0 | 6 | 319 | 60 | 0·75 | $6/1\cdot91\times10^4$ | |
| | | 463 | 51 | 0·65 | $5/2\cdot36\times10^4$ | $2\cdot95\pm0\cdot44$ |
| | | 401 | 65 | 0·72 | $20/2\cdot61\times10^4$ | |
| 2·0 | 6 | 194 | 37 | 0·26 | $4/0\cdot72\times10^4$ | |
| | | 368 | 76 | 0·30 | $12/2\cdot80\times10^4$ | $4\cdot50\pm0\cdot46$ |
| | | 525 | 60 | 0·34 | $15/3\cdot15\times10^4$ | |
| | | 254 | 70 | 0·25 | $8/1\cdot78\times10^4$ | |
| (combined controls) | | 435 | 475 | 0·18* | $0/2\cdot07\times10^5$ | 0 |

* Plating efficiency.

transformation rate is less than $10^{-6}$. The rate of transformation clearly increases with drug concentration. On the other hand, the period for which the drug is in contact with the cells (3 or 6 days) appears to have little influence on the number of cells transformed. It is relevant and interesting to compare the potential carcinogenicity of MIS with other known carcinogens, such as X-rays. Fig. 4a shows the relationship between transformation and X-ray dose; the number of transformants per surviving cell increases with dose, but approaches a plateau at a frequency of about $2 \times 10^{-3}$ for doses over 6 Gy. By comparing Panels (a) and (b) of Fig. 4 it is possible to calculate the drug concentrations that are equivalent to various doses of X-rays in their ability to induce transformation. This "equivalence" is summarized in Fig. 5, where the dose of radiation is plotted against the concentration of MIS which results in the same frequency of transformation.

The results of experiments in which MIS was combined with X-rays are summarized in Table II and plotted in Fig. 6. The combination of drug *and* radiation resulted in a significant increase in the frequency of transformations compared with X-rays or drug alone. The drug treatment was a 3-day exposure to a concentration of 1 mM, beginning 24 h before irradiation and continuing for 48 h afterwards. Three doses of X-rays were used, 1, 2 and 4 Gy.

### DISCUSSION

Misonidazole promises to be a valuable

FIG. 4.—The proportion of C3H/10T½ cells transformed (a) as a function of X-ray dose. (b) as a function of concentration of MIS for 3 days (○) or 6 days (●).

FIG. 5.—A comparison of the dose of X-rays (Gy) and the concentration of MIS (for 3 days) required to produce an equal incidence of transformation in C3H/10T½ cells.

TABLE II.—*Frequency of transformation (transformants per* $10^4$ *surviving cells)*

| Treatment | MIS (3-day exposure) | X-ray alone | Combination MIS+X-rays* | |
|---|---|---|---|---|
| 1 mM MIS and/or 1 Gy X-rays | 2·12 | 2·35 | 6·21 | (0·33)* |
| | 2·94 | 1·90 | 4·53 | (0·36) |
| | 2·53±0·41 | 2·13±0·23 | 5·37±0·84 | (0·35) |
| 1 mM MIS and/or 2 Gy X-rays | 3·41 | 3·87 | 15·0 | (0·15) |
| | 2·94 | 2·56 | 8·51 | (0·25) |
| | 3·18±0·24 | 3·22±0·23 | 11·8±3·2 | (0·20) |
| 1 mM MIS and/or 4 Gy X-rays | 2·12 | 9·48 | 20·0 | (0·16) |
| | 2·94 | 7·36 | 12·3 | (0·19) |
| | 2·53±0·41 | 8·42±1·06 | 16·2±3·9 | (0·18) |

* In parentheses, surviving fraction.

FIG. 6.—The proportion of C3H/10T½ cells transformed by various doses of X-rays, with and without MIS (1 mM) for 3 days.

addition to the armamentarium of the radiotherapist because it sensitizes hypoxic cells which are otherwise relatively resistant to X-rays and may limit the radiocurability of some human tumours (Asquith *et al.*, 1974; Denekamp and Harris, 1975; Thomlinson and Gray, 1955). MIS may also find a place in multidrug chemotherapy regimens, because it is also cytotoxic towards hypoxic cells (Hall *et al.*, 1977, Hall and Roizin-Towle, 1975; Stratford and Adams, 1977).

These undoubted benefits must be weighed against the possibility that the nitroimidazoles are potent carcinogens. When MIS is used clinically as a sensitizer in conjunction with radiotherapy, serum levels in the range 0·5 to 1 mM can be achieved. The maximum serum concentration occurs 3 to 4 h after oral administrations of the drug, and subsequently decays exponentially with a half-life of ~12 h. Based on Fig. 5, this drug treatment carried with it a carcinogenic potential equal to an X-ray dose of about 1 Gy. It is pertinent to note that when radiation is used as a therapeutic modality, the high dose is confined to the tumour, its immediate surroundings and the necessary transit normal tissues. By contrast, when MIS is used clinically it comes into contact with virtually *all* the normal tissues of the body.

The experiments combining MIS and X-rays showed that the combination produces a significant increase in the frequency of transformations compared with either the drug or the radiation alone. It would clearly be of interest to know whether the interaction between the two agents is additive or synergistic. The drug treatment alone (1 mM for 3 days) resulted in a transformation frequency of about $2{\cdot}5\times10^{-4}$. A dose of 1 Gy alone produced a transformation frequency of about $2{\cdot}1\times10^{-4}$. The combination of 1 Gy plus drug resulted in a frequency of about $5{\cdot}2 \times 10^{-4}$, which is not significantly different from the sum of the individual contributions of the two modalities ($2{\cdot}5+2{\cdot}1=4{\cdot}6$) which would suggest additivity between radiation and drug. However, when 2 Gy of X-rays was combined with the drug treatment, the transformation frequency produced ($1{\cdot}3 \times 10^{-3}$) was significantly greater than the sum of the frequencies produced by drug and radiation alone ($2{\cdot}5+3{\cdot}2= 5{\cdot}7 \times 10^{-4}$) which suggests synergism in the action of drug and radiation. It is difficult to draw any conclusions from the highest radiation dose used, because the transformation frequency produced by 4 Gy+drug approaches the plateau of about $2 \times 10^{-3}$, which appears never to be exceeded by either radiation or drug treatments. The present data, therefore, clearly show that combining radiation and MIS results in more transformations than either agent alone, but is equivocal on the question of additivity *vs* synergism.

These studies with an *in vitro* transformation system have shown that the new generation of hypoxic cell sensitizers have as great a potential as radiation for producing oncogenic transformation. They highlight the need for further investigations with more complex animal model systems. When new drugs such as MIS have passed their early clinical tests and are used in younger patients with less advanced tumours, who therefore have a longer life expectancy, the possibility of inducing a second neoplasm while treating the first can no longer be ignored.

This investigation was supported by Contract EP-78-S-02-4733 from the Department of Energy and Grants Number CA-12536 and CA-18506 awarded by the National Cancer Institute, DHEW. The drug Misonidazole was generously supplied by the Roche Company, and we gladly acknowledge much fruitful discussion with Professor G. E. Adams n the design of this investigation.

### REFERENCES

ADAMS, G. E. (1973) Chemical radiosensitization of hypoxic cells. *Br. Med. Bull.*, **29**, 48.

ADAMS, G. E. & DEWEY, D. L. (1963) Hydrated electrons and radiobiological sensitization. *Biochim. Biophys. Res. Commun.*, **12**, 473.

ASQUITH, J. C., WATTS, M. E., PATEL, K., SMITHEN, C. E. & ADAMS, G. E. (1974) Electron affinic sensitization *v.* radiosensitization of hypoxic bacteria and mammalian cells *in vitro* by some nitroimidazoles and nitropyrazoles. *Radiat. Res.*, **60**, 108.

BROWN, J. M. (1975) Selective radiosensitization of the hypoxic cells of mouse tumors with the nitroimidazoles metronidazole and Ro-07-0582. *Radiat. Res.*, **64**, 633.

CHAPMAN, J. D., REUVERS, A. P., BORSA, J. & GREENSTOCK, C. L. (1973) Chemical radioprotection and radiosensitization of mammalian cells growing *in vitro*. *Radiat. Res.*, **56**, 291.

CONNOR, T. H., STOECKEL, M., EVRARD, J. & LEGATOR, M. S. (1977) The contribution of metronidazole and two metabolites to the mutagenic activity detected in urine of treated humans and mice. *Cancer Res.*, **37**, 629.

DENEKAMP, J. & HARRIS, S. R. (1975) Tests of two electron-affinic radiosensitizers *in vivo* using regrowth of an experimental carcinoma. *Radiat. Res.*, **61**, 191.

FOSTER, J. L., CONROY, P. J., SEARLE, A. J. & Willson, R. L. (1976) Metronidazole (Flagyl): characterization as a cytotoxic drug specific for hypoxic tumour cells. *Br. J. Cancer*, **33**, 486.

GRAY, A. J., DISCHE, S., ADAMS, G. E., FLOCKHART, I. R. & FOSTER, J. L. (1976) Clinical testing of the radiosensitizer Ro-07-0582. I. Dose tolerance, serum and tumour concentrations. *Clin. Radiol.*, **27**, 151.

HALL, E. J., ASTOR, M., GEARD, C. & BIAGLOW, J. (1977) Cytoxicity of Ro-07-0582: enhancement by hyperthermia and protection by cysteine. *Br. J. Cancer*, **35**, 809.

HALL, E. J. & ROIZIN-TOWLE, L. (1975) Hypoxic sensitizers: radiobiological studies at the cellular level. *Radiology*, **117**, 453.

REZNIKOFF, C. A., BRANKOW, D. W. & HEIDELBERGER, C. (1973*a*) Establishment of C3H mouse embryo cells. *Cancer Res.*, **33**, 3231.

REZNIKOFF, C. A., BRANKOW, D. W., HEIDELBERGER, C. (1973*b*) Quantitative studies of chemical transformation of C3H mouse embryo cells. *Cancer Res.*, **33**, 3239.

SHELDON, P. W., FOSTER, J. L. & FOWLER, J. F. (1974) Radiosensitization of C3H mouse mammary tumours by a 2-nitroimidazole drug. *Br. J. Cancer*, **30**, 560.

SPECK, W. T., STEIN, A. B. & ROSENKRANZ, H. S. (1976) Mutagenicity of metronidazole: presence of several active metabolites in human urine. *J. Natl. Cancer Inst.*, **56**, 283.

STRATFORD, I. J. & ADAMS, G. E. (1977) Effect of hyperthermia on differential cytoxicity of a hypoxic cell radiosensitizer, Ro-07-0582, on mammalian cells *in vitro*. *Br. J. Cancer*, **35**, 309.

THOMLINSON, R. H. & GRAY, L. H. (1955) The histological structure of some human lung cancers and the possible implications of radiotherapy. *Br. J. Cancer*, **9**, 539.

VOOGD, C. E., VAN DER STEL, J. J. & JACOBS, J. A. (1974) The mutagenic action of nitroimidazoles. I. Metronidazole, nimoragole, dimetridazole and ronidazole. *Mutat. Res.*, **26**, 483.